# Professor Breckinridge’s Address

**Published:** 1854-04

**Authors:** 


### PROFESSOR BRECKINRIDGE'S ADDRESS.

---

We have received from our friend, Professor R. J. BRECKINRIDGE, a copy of his valedictory address. The subject is *Medicine as a Science and an Art*, the claims of which to the respect and gratitude of men, in both its aspects, are clearly and ably set forth.

Without claiming to be perfect either as a science or an art, and, perhaps, with slender prospects of attaining to that point long before the millennium, Dr. B., nevertheless makes it evident, that it has been a precious boon to our afflicted race. The concluding thoughts of this address are just and important. It is urged upon the young men to whom it was delivered, that all depended upon themselves whether they were to be cyphers or shining lights in their profession—that to become *great* physicians was impossible, "without continuous labor, ceaseless diligence, and untiring perseverance." Nothing is more true. It is application, diligent, unwearied, persevering, that secures eminence in our profession.

